# Author Correction: Obesity and ethnicity alter gene expression in skin

**DOI:** 10.1038/s41598-021-87276-x

**Published:** 2021-03-31

**Authors:** Jeanne M. Walker, Sandra Garcet, Jose O. Aleman, Christopher E. Mason, David Danko, Daniel Butler, Simone Zuffa, Jonathan R. Swann, James Krueger, Jan L. Breslow, Peter R. Holt

**Affiliations:** 1 The Rockefeller University Hospital, New York, NY 10065 USA; 2 Laboratory of Investigational Dermatology, The Rockefeller University, New York, NY 10065 USA; 3 Laboratory of Biochemical Genetics and Metabolism, The Rockefeller University, New York, NY 10065 USA; 4 Laboratory of Translational Obesity Research, New York University Langone Health, New York, NY 10016 USA; 5 Weill Cornell Medical College, New York, NY 10065 USA; 6 Department of Metabolism, Digestion, and Reproduction, Imperial College London, London, UK; 7 School of Human Development and Health, Faculty of Medicine, University of Southampton, Southampton, UK

Correction to: *Scientific Reports* 10.1038/s41598-020-70244-2, published online 21 August 2020

Daniel Butler was omitted from the author list in the original version of this Article.

The Author contributions section now reads:

“J.M.W. designed, conducted, and contributed to the writing of the manuscript, prepared Fig. 1. S.G. evaluated and did statistical analysis on the skin and fat samples, prepared Figs. 2–9. J.O.A. evaluated and contributed to writing the manuscript. D.B prepared and sequenced DNA libraries for the skin microbiota data, and wrote the applicable parts of the methods section. C.M. analyzed and wrote up the skin microbiota data, prepared Fig. 10. All authors have read the manuscript and approved its contents. D.D. analyzed and wrote up the skin microbiota data. S.Z. ran and analyzed the skin metabolite data. J.S. assisted in design, analysis and wrote up the skin metabolite data. J.K. assisted in analysis write up of skin and fat data. J.L.B. assisted in analysis, interpretation and writing of the manuscript. P.R.H. designed, analyzed, interpreted the data, and was the primary author of the manuscript.”

This has been corrected in the PDF and HTML versions of the Article, and in the accompanying Supplementary Information file.

