# Supplementary material for: Obesity and ethnicity alter gene expression in skin
Source: Sci Rep. 2020 Aug 21;10:14079. doi: 10.1038/s41598-020-70244-2 (PMC7442822; doi:10.1038/s41598-020-70244-2)
Supplement: Supplementary file 1 — Supplementary information [file 41598_2020_70244_MOESM1_ESM.pdf]

Supplements

Obesity and Ethnicity Alter Gene Expression in Skin

Jeanne M. Walker, Sandra Garcet, Jose O. Aleman, Christopher E. Mason, David Danko,

Daniel Butler, Simone Zuffa, Jonathan Swann, James Krueger, Jan L. Breslow, Peter R. Holt

Table S1

Anthropometric Measurements

	Obese Cohort n=10		Non-obese Cohort n=10		
	Mean (+/-SD)	Range	Mean (+/-SD)	Range	*p*
Age (years)	58.8 (5.8)	48-69	60.2 (5.9)	49-69	0.556
Weight (kg)	109 (9.9)	93.4-126	59.4 (8)	49.4-71.1	0.000
BMI (kg/m^2)	40.7 (4.1)	35.3-47.3	22.4 (2.7)	18.7-26	0.000
Waist (cm)	117 (7.4)	104-128	80.4 (10)	66-98.5	0.000
Hip (cm)	133 (7.6)	124-148	96 (9.3)	86-115	0.000
Waist/hip ratio	0.88 (0.05)	0.82-0.9	0.84 (0.06)	0.77-0.94	0.120

Comparison of anthropometric measurements in the 10 obese and 10 non-obese cohorts mean +/- standard deviation.

Supplemental Tables: Genes

Table S2: **Skin: Obese vs Non-obese 1st 70 genes**

SYMBOL	Normal.Weight	Obese	lgFCH_Obese.vs.Normal.Weight	FCH_Obese.vs.Normal.Weight	pvals_Obese.vs.Normal.Weight	fdrs_Obese.vs.Normal.Weight	StatusFCH1.5FDR0.1_Obese.vs.Normal.Weight
S100A7A	5.99	7.77	1.78	3.44	0.00	0.08	1.00
DEFB4A	7.84	9.48	1.64	3.11	0.00	0.07	1.00
PRG4	4.63	6.03	1.40	2.63	0.00	0.09	1.00
CORIN	7.63	8.93	1.30	2.46	0.00	0.03	1.00
CORIN	6.37	7.43	1.06	2.09	0.00	0.08	1.00
CNTNAP3	5.86	6.87	1.01	2.02	0.00	0.10	1.00
C1QTNF3	5.62	6.56	0.95	1.93	0.00	0.03	1.00
COL12A1	5.29	6.18	0.89	1.86	0.00	0.10	1.00
MORF4L2	5.84	6.72	0.88	1.85	0.00	0.10	1.00
FMO3	5.25	6.11	0.85	1.80	0.00	0.09	1.00
SLC16A10	8.81	9.65	0.84	1.79	0.00	0.08	1.00
CORIN	6.78	7.62	0.84	1.79	0.00	0.08	1.00
LINC01091	7.09	7.90	0.80	1.75	0.00	0.06	1.00
FAM134B	7.24	8.02	0.77	1.71	0.00	0.08	1.00
SPRR2C	4.83	5.59	0.77	1.70	0.00	0.06	1.00
CAPZA2	4.02	4.73	0.70	1.63	0.00	0.07	1.00
WDR11	7.76	8.46	0.70	1.63	0.00	0.08	1.00
PPP2R3A	9.38	10.07	0.69	1.62	0.00	0.10	1.00
TRAK1	8.54	9.23	0.70	1.62	0.00	0.06	1.00
PTPRK	6.03	6.70	0.68	1.60	0.00	0.07	1.00
EIF3M	7.15	7.82	0.67	1.59	0.00	0.09	1.00

Table S2 cont'd

**RHOA**	5.84	6.49	0.65	1.57	0.00	0.08	1.00
**TRAK1**	5.69	6.34	0.65	1.56	0.00	0.03	1.00
**P2RY1**	6.11	6.74	0.63	1.55	0.00	0.10	1.00
**ASPM**	5.86	6.49	0.63	1.55	0.00	0.05	1.00
**RAB27B**	6.88	7.50	0.63	1.54	0.00	0.02	1.00
**NRIP1**	6.70	7.32	0.61	1.53	0.00	0.08	1.00
**WASL**	8.31	8.92	0.61	1.53	0.00	0.04	1.00
**TRPC6**	6.81	7.42	0.61	1.53	0.00	0.02	1.00
**ATP10B**	7.92	8.53	0.61	1.53	0.00	0.10	1.00
**HIST1H2BC**	6.34	6.94	0.60	1.52	0.00	0.02	1.00
**HECTD4**	6.34	6.93	0.59	1.51	0.00	0.09	1.00
**LOC284930**	7.64	8.23	0.59	1.51	0.00	0.03	1.00
**BMP2**	7.56	8.15	0.59	1.51	0.00	0.10	1.00
**EZR**	9.10	9.69	0.59	1.51	0.00	0.06	1.00
**EML6**	6.07	6.67	0.60	1.51	0.00	0.10	1.00
**ARHGAP44**	8.53	7.94	-0.59	-1.50	0.00	0.08	-1.00
**LINC00950**	6.20	5.61	-0.59	-1.50	0.00	0.03	-1.00
**MYO5C**	10.53	9.94	-0.59	-1.50	0.00	0.06	-1.00
**KLHDC9**	6.25	5.66	-0.59	-1.50	0.00	0.05	-1.00
**LOC441242**	5.43	4.84	-0.59	-1.50	0.00	0.06	-1.00
**RASL10A**	5.72	5.13	-0.59	-1.51	0.00	0.09	-1.00
**GGTLC1**	7.82	7.23	-0.59	-1.51	0.00	0.07	-1.00
**SPDEF**	7.39	6.79	-0.60	-1.51	0.00	0.08	-1.00
**MRAS**	9.78	9.19	-0.59	-1.51	0.00	0.09	-1.00
**FAM27E3**	6.73	6.13	-0.60	-1.51	0.00	0.10	-1.00
**ENPP5**	8.22	7.62	-0.60	-1.51	0.00	0.07	-1.00
**SCAND2P**	8.53	7.93	-0.59	-1.51	0.00	0.05	-1.00
**DOK6**	5.94	5.35	-0.60	-1.51	0.00	0.10	-1.00
**SOCS2**	6.45	5.86	-0.59	-1.51	0.00	0.05	-1.00
**SGSM2**	10.97	10.37	-0.60	-1.51	0.00	0.07	-1.00
**BRE-AS1**	4.59	3.99	-0.61	-1.52	0.00	0.09	-1.00
**REEP1**	8.44	7.84	-0.60	-1.52	0.00	0.05	-1.00
**CYP2C9**	6.59	5.99	-0.61	-1.52	0.00	0.06	-1.00
**ZBED8**	7.70	7.10	-0.60	-1.52	0.00	0.10	-1.00
**CPAMD8**	6.00	5.39	-0.61	-1.52	0.00	0.04	-1.00
**DNAH1**	8.96	8.35	-0.61	-1.52	0.00	0.10	-1.00

Table S3

Skin: Obese vs Non-obese last 70 genes

SYMBOL	Normal.Weight	Obese	lgFCH_Obese.vs.Normal.Weight	FCH_Obese.vs.Normal.Weight	pvals_Obese.vs.Normal.Weight	fdrs_Obese.vs.Normal.Weight	StatusFCH1.5FDR0.1_Obese.vs.Normal.Weight
SHANK2	7.03	5.80	-1.23	-2.35	0.00	0.04	-1.00
TOX3	5.95	4.69	-1.26	-2.39	0.00	0.04	-1.00
PPP1R1B	9.26	7.99	-1.26	-2.40	0.00	0.03	-1.00
STAC2	8.49	7.23	-1.26	-2.40	0.00	0.05	-1.00
MEGF10	7.01	5.73	-1.28	-2.42	0.00	0.08	-1.00
SH3GL2	4.79	3.51	-1.29	-2.44	0.00	0.06	-1.00
EPHA7	7.42	6.14	-1.29	-2.44	0.00	0.07	-1.00
FUT9	4.78	3.47	-1.31	-2.47	0.00	0.05	-1.00
CLCNKB	8.28	6.96	-1.32	-2.49	0.00	0.05	-1.00
SFRP1	11.51	10.19	-1.32	-2.50	0.00	0.07	-1.00
MMP7	9.51	8.19	-1.32	-2.50	0.00	0.05	-1.00
KCNK5	9.64	8.31	-1.33	-2.51	0.00	0.08	-1.00
ATP6V1B1	7.94	6.60	-1.34	-2.53	0.00	0.04	-1.00
B3GALT5	8.78	7.44	-1.34	-2.54	0.00	0.07	-1.00
KRT18	10.06	8.71	-1.35	-2.55	0.00	0.02	-1.00
CEACAM6	8.96	7.60	-1.36	-2.56	0.00	0.05	-1.00
F5	6.91	5.54	-1.36	-2.57	0.00	0.07	-1.00
CEACAM5	8.21	6.84	-1.37	-2.58	0.00	0.09	-1.00
SLC34A2	5.87	4.50	-1.37	-2.59	0.00	0.08	-1.00
ROPN1	5.68	4.31	-1.37	-2.59	0.00	0.02	-1.00
GABRP	9.40	8.01	-1.39	-2.63	0.00	0.10	-1.00
TTC6	5.49	4.06	-1.42	-2.68	0.00	0.10	-1.00

Table S3 cont'd

**SFRP1**	8.81	7.35	-1.46	-2.75	0.00	0.03	-1.00
**UGT8**	7.07	5.59	-1.48	-2.79	0.00	0.06	-1.00
**PRR4**	9.65	8.16	-1.49	-2.81	0.00	0.08	-1.00
**CFTR**	8.08	6.58	-1.50	-2.82	0.00	0.07	-1.00
**SPX**	4.39	2.88	-1.51	-2.84	0.00	0.06	-1.00
**VTCN1**	6.86	5.35	-1.52	-2.86	0.00	0.04	-1.00
**GRIA2**	5.80	4.27	-1.53	-2.88	0.00	0.10	-1.00
**RHPN2**	9.59	8.05	-1.54	-2.91	0.00	0.09	-1.00
**OBP2B**	7.33	5.79	-1.54	-2.91	0.00	0.03	-1.00
**LMF1**	8.43	6.88	-1.55	-2.93	0.00	0.04	-1.00
**GLYATL2**	7.26	5.71	-1.55	-2.94	0.00	0.05	-1.00
**ATP6V0A4**	10.27	8.70	-1.58	-2.98	0.00	0.08	-1.00
**SHANK2**	7.41	5.82	-1.59	-3.01	0.00	0.04	-1.00
**LMF1**	7.55	5.95	-1.60	-3.03	0.00	0.04	-1.00
**CEACAM1**	7.42	5.81	-1.60	-3.04	0.00	0.06	-1.00
**PADI2**	10.32	8.71	-1.61	-3.05	0.00	0.04	-1.00
**ELF5**	10.17	8.56	-1.61	-3.06	0.00	0.07	-1.00
**MB**	7.79	6.16	-1.62	-3.08	0.00	0.03	-1.00
**PIGR**	7.70	6.08	-1.62	-3.08	0.00	0.09	-1.00
**ADCY8**	7.31	5.68	-1.63	-3.09	0.00	0.02	-1.00
**IREB2**	5.97	4.31	-1.65	-3.14	0.00	0.09	-1.00
**CHRM3**	6.77	5.12	-1.65	-3.14	0.00	0.02	-1.00
**MYBPC1**	8.26	6.57	-1.69	-3.23	0.00	0.07	-1.00
**OBP2B**	6.92	5.22	-1.69	-3.23	0.00	0.05	-1.00
**TMPRSS2**	8.28	6.56	-1.72	-3.29	0.00	0.02	-1.00
**ROPN1**	8.63	6.90	-1.73	-3.32	0.00	0.06	-1.00
**ROPN1**	8.43	6.69	-1.74	-3.34	0.00	0.05	-1.00
**KRT7**	6.91	5.15	-1.76	-3.38	0.00	0.03	-1.00
**ROPN1**	8.15	6.39	-1.76	-3.39	0.00	0.06	-1.00
**SLC26A7**	7.04	5.21	-1.83	-3.57	0.00	0.07	-1.00
**GRIA2**	6.47	4.63	-1.85	-3.59	0.00	0.07	-1.00
**DNER**	10.02	8.17	-1.85	-3.61	0.00	0.08	-1.00
**GRB14**	9.20	7.34	-1.86	-3.63	0.00	0.06	-1.00
**FOXI1**	6.78	4.91	-1.87	-3.65	0.00	0.03	-1.00
**KRT7**	8.87	7.01	-1.87	-3.65	0.00	0.03	-1.00
**KRT19**	9.64	7.75	-1.89	-3.71	0.00	0.05	-1.00
**TMEM213**	7.92	6.03	-1.90	-3.72	0.00	0.10	-1.00
**PIGR**	8.43	6.52	-1.90	-3.74	0.00	0.08	-1.00
**KIAA1324**	9.14	7.21	-1.93	-3.81	0.00	0.02	-1.00
**CHRM3**	7.90	5.95	-1.95	-3.86	0.00	0.03	-1.00
**KIAA1324**	8.71	6.75	-1.95	-3.87	0.00	0.09	-1.00

Table S3 cont'd

**MFI2**	8.11	6.00	-2.11	-4.33	0.00	0.03	-1.00
**NELL1**	7.11	4.91	-2.20	-4.61	0.00	0.03	-1.00
**CLDN10**	9.84	7.58	-2.26	-4.81	0.00	0.07	-1.00
**KRT19**	10.66	8.35	-2.31	-4.96	0.00	0.03	-1.00
**SLC14A1**	8.95	6.59	-2.36	-5.15	0.00	0.05	-1.00
**PROM1**	9.21	6.72	-2.50	-5.65	0.00	0.04	-1.00
**AQP5**	9.66	7.12	-2.54	-5.81	0.00	0.07	-1.00
**CRISP3**	9.12	6.54	-2.57	-5.95	0.00	0.06	-1.00

Table S4

Fat: Obese vs Non-obese 1st 70 genes

SYMBOL	Normal.Weight	Obese	lgFCH_Obese.vs.Normal.Weight	FCH_Obese.vs.Normal.Weight	pvals_Obese.vs.Normal.Weight	fdrs_Obese.vs.Normal.Weight	StatusFCH1.5FDR0.1_Obese.vs.Normal.Weight
SPP1	5.04	8.57	3.53	11.52	0.00	0.00	1.00
SPP1	6.89	10.05	3.16	8.96	0.00	0.00	1.00
EGFL6	8.32	11.40	3.09	8.51	0.00	0.01	1.00
UNC13C	4.14	7.05	2.91	7.54	0.00	0.00	1.00
MMP9	5.61	8.43	2.82	7.07	0.00	0.00	1.00
SERPINB3	5.05	7.86	2.81	7.00	0.00	0.00	1.00
IL1RN	6.47	9.25	2.78	6.86	0.00	0.00	1.00
SPRR1A	3.60	6.31	2.71	6.56	0.00	0.00	1.00
PLA2G7	5.49	8.14	2.66	6.30	0.00	0.00	1.00
SERPINB3	3.99	6.64	2.64	6.25	0.00	0.00	1.00
S100A7A	5.05	7.64	2.59	6.01	0.00	0.00	1.00
THBS1	6.60	9.14	2.54	5.83	0.00	0.00	1.00
SPRR1B	6.24	8.78	2.54	5.82	0.00	0.00	1.00
CHI3L1	6.45	8.96	2.51	5.69	0.00	0.00	1.00
SERPINB4	4.85	7.34	2.50	5.64	0.00	0.00	1.00
DEFB4A	7.36	9.71	2.35	5.09	0.00	0.00	1.00
KRTDAP	6.57	8.86	2.29	4.88	0.00	0.00	1.00
SPRR3	6.34	8.62	2.28	4.86	0.00	0.00	1.00
LCE3D	4.89	7.15	2.25	4.77	0.00	0.01	1.00
SPRR2G	6.35	8.56	2.21	4.63	0.00	0.00	1.00
CHI3L1	6.11	8.29	2.18	4.52	0.00	0.00	1.00
UNC13C	3.12	5.29	2.17	4.51	0.00	0.00	1.00

Table S4 cont'd

COMP	5.29	7.46	2.17	4.49	0.00	0.02	1.00
LAMP3	4.26	6.41	2.15	4.45	0.00	0.00	1.00
PI3	6.50	8.65	2.15	4.42	0.00	0.00	1.00
ABCC3	5.99	8.11	2.12	4.34	0.00	0.00	1.00
LINC00968	7.00	9.12	2.12	4.34	0.00	0.00	1.00
ACTC1	5.93	8.04	2.11	4.33	0.02	0.10	1.00
SCIN	5.89	7.99	2.10	4.29	0.00	0.02	1.00
PI3	6.89	8.99	2.10	4.29	0.00	0.00	1.00
TNC	4.83	6.91	2.07	4.21	0.00	0.02	1.00
COL11A1	5.54	7.62	2.08	4.21	0.00	0.00	1.00
DCSTAMP	3.62	5.69	2.06	4.18	0.00	0.00	1.00
S100A7	9.20	11.24	2.05	4.13	0.00	0.00	1.00
TNC	4.71	6.74	2.03	4.08	0.00	0.01	1.00
FCGR1B	5.15	7.17	2.02	4.07	0.00	0.00	1.00
IGLV1-44	3.77	5.78	2.01	4.04	0.00	0.00	1.00
SPRR2D	6.41	8.38	1.97	3.92	0.00	0.01	1.00
FCGBP	6.32	8.29	1.96	3.90	0.00	0.00	1.00
CCL22	5.19	7.15	1.96	3.88	0.00	0.00	1.00
NPR3	7.36	9.29	1.94	3.83	0.00	0.02	1.00
TNC	8.26	10.18	1.92	3.79	0.00	0.02	1.00
UBE2QL1	5.06	6.98	1.92	3.79	0.00	0.00	1.00
CXCL8	4.88	6.75	1.87	3.66	0.00	0.00	1.00
IGLC1	5.11	6.97	1.86	3.62	0.00	0.01	1.00
POPDC3	3.51	5.37	1.85	3.62	0.00	0.01	1.00
PPP2R2C	5.59	7.44	1.85	3.61	0.01	0.04	1.00
IGH	6.90	8.74	1.84	3.58	0.00	0.01	1.00
SELE	4.55	6.38	1.83	3.57	0.00	0.00	1.00
CLEC7A	5.22	7.05	1.83	3.55	0.00	0.00	1.00
UCHL1	9.50	11.33	1.83	3.55	0.00	0.00	1.00
LOC101929398	6.14	7.96	1.82	3.53	0.00	0.01	1.00
	5.47	6.30	0.83	1.78	0.01	0.06	1.00
TREM2	4.77	6.59	1.82	3.52	0.00	0.00	1.00
THBS1	8.02	9.84	1.81	3.51	0.00	0.03	1.00
FCGR1A	5.88	7.66	1.78	3.44	0.00	0.00	1.00
P2RX1	6.94	8.72	1.77	3.42	0.01	0.07	1.00
RGS1	3.53	5.30	1.77	3.42	0.00	0.00	1.00
CCL3	5.45	7.21	1.76	3.40	0.00	0.00	1.00
SERPINB13	3.15	4.92	1.77	3.40	0.00	0.02	1.00
FCGR1A	3.70	5.46	1.76	3.39	0.00	0.00	1.00
SCIN	5.05	6.80	1.75	3.36	0.00	0.03	1.00
SBSN	4.90	6.65	1.74	3.35	0.00	0.01	1.00

Table S4 cont'd

**SPRR1A**	6.31	8.03	1.72	3.30	0.00	0.00	1.00
**DIO2**	4.88	6.60	1.72	3.30	0.01	0.05	1.00
**CEP55**	4.67	6.37	1.70	3.25	0.00	0.00	1.00
**ADAMDEC1**	2.82	4.52	1.69	3.24	0.00	0.00	1.00
**KRT6A**	6.14	7.84	1.70	3.24	0.01	0.06	1.00
**ARG1**	3.55	5.23	1.68	3.21	0.00	0.02	1.00
**TRAT1**	5.04	6.71	1.67	3.18	0.00	0.00	1.00
**RRM2**	4.34	6.00	1.66	3.15	0.00	0.00	1.00
**ACTA1**	4.24	5.90	1.65	3.14	0.01	0.07	1.00

Table S5

Fat: Obese vs Non-obese last 70 genes

SYMBOL	Normal.Weight	Obese	lgFCH_Obese.vs.Normal.Weight	FCH_Obese.vs.Normal.Weight	pvals_Obese.vs.Normal.Weight	fdrs_Obese.vs.Normal.Weight	StatusFCH1.5FDR0.1_Obese.vs.Normal.Weight
GYS2	8.05826	6.581453	-1.48	-2.78	0.00719	0.0527	-1
TSHR	5.955878	4.452574	-1.5	-2.83	0.0129	0.0748	-1
CHODL	5.449809	3.948163	-1.5	-2.83	4.01E-05	0.00286	-1
	3.709156	4.413575	0.7	1.63	0.000357	0.0091	1
CPNE4	6.713133	5.211148	-1.5	-2.83	0.000344	0.00889	-1
CYP4F29P	7.56177	6.055927	-1.51	-2.84	0.000349	0.00897	-1
CIDEA	10.59601	9.087702	-1.51	-2.84	0.000097	0.00447	-1
SYT17	7.402136	5.889865	-1.51	-2.85	7.02E-05	0.00373	-1
	6.27391	5.569122	-0.7	-1.63	8.51E-06	0.00129	-1
RASL10B	6.434846	4.914091	-1.52	-2.87	5.52E-05	0.00334	-1
	5.013853	5.999627	0.99	1.98	0.00378	0.0357	1
	3.92753	4.588919	0.66	1.58	0.0111	0.0684	1
WDR86	7.550435	6.009443	-1.54	-2.91	9.19E-05	0.00432	-1
NAALAD2	9.806165	8.25292	-1.55	-2.93	1.95E-08	0.000132	-1
	7.778574	6.502444	-1.28	-2.42	0.00137	0.0196	-1
CPNE4	5.444387	3.878195	-1.57	-2.96	0.000804	0.0143	-1
GLUL	10.897	9.325681	-1.57	-2.97	4.56E-07	0.000442	-1
RGS3	10.13124	8.559374	-1.57	-2.97	4.61E-07	0.000442	-1
CAPN6	7.416375	5.839098	-1.58	-2.98	2.47E-07	0.000379	-1
	4.492622	5.168973	0.68	1.6	0.00278	0.0296	1
GSDMB	9.557125	7.974478	-1.58	-3	5.95E-08	0.000205	-1
CECR2	7.489781	5.869481	-1.62	-3.07	0.00141	0.0199	-1
	5.980785	6.804565	0.82	1.77	0.000891	0.0152	1
	2.967631	3.555909	0.59	1.5	2.38E-05	0.00217	1
	4.405341	3.332135	-1.07	-2.1	0.00353	0.0344	-1

Table S5 cont'd							
CETP	8.405207	6.779649	-1.63	-3.09	0.000457	0.0105	-1
	7.019169	6.390175	-0.63	-1.55	0.019	0.0948	-1
AZGP1	11.80998	10.16363	-1.65	-3.13	1.03E-05	0.00142	-1
	6.50	5.76	-0.75	-1.68	0.00	0.00	-1.00
**STOX1**	9.73	8.08	-1.65	-3.13	0.00	0.00	-1.00
**ALPK3**	9.09	7.43	-1.65	-3.14	0.00	0.00	-1.00
**APOB**	10.27	8.61	-1.66	-3.17	0.00	0.00	-1.00
**ALPK3**	9.18	7.51	-1.67	-3.18	0.00	0.00	-1.00
	7.61	8.30	0.69	1.61	0.00	0.01	1.00
**GLUL**	9.84	8.16	-1.68	-3.20	0.00	0.00	-1.00
**OK/SW-CL.36**	4.99	3.28	-1.71	-3.27	0.00	0.01	-1.00
**DMRT2**	8.88	7.16	-1.72	-3.28	0.00	0.01	-1.00
**LOC105376379**	6.04	4.32	-1.71	-3.28	0.00	0.01	-1.00
	3.63	4.34	0.71	1.64	0.01	0.05	1.00
	4.58	5.61	1.03	2.04	0.00	0.01	1.00
**TOX3**	5.68	3.95	-1.73	-3.32	0.02	0.08	-1.00
**EYA1**	6.63	4.89	-1.74	-3.35	0.00	0.00	-1.00
	6.54	5.71	-0.83	-1.78	0.00	0.01	-1.00
**TOX3**	5.84	4.03	-1.80	-3.49	0.01	0.07	-1.00
**FCN2**	6.61	4.76	-1.86	-3.62	0.00	0.01	-1.00
	4.94	5.54	0.60	1.51	0.00	0.00	1.00
	7.65	8.62	0.97	1.96	0.00	0.01	1.00
**COL6A6**	6.36	4.51	-1.86	-3.63	0.00	0.00	-1.00
**AZGP1**	11.30	9.42	-1.88	-3.67	0.00	0.00	-1.00
**LOC90768**	7.53	5.65	-1.88	-3.68	0.00	0.00	-1.00
	6.05	6.91	0.87	1.82	0.00	0.03	1.00
**ALDH1L1-AS2**	7.54	5.66	-1.89	-3.70	0.00	0.00	-1.00
**LDHD**	8.00	6.11	-1.89	-3.71	0.00	0.00	-1.00
**GLUL**	8.81	6.92	-1.89	-3.72	0.00	0.00	-1.00
**SPX**	11.14	9.17	-1.97	-3.92	0.00	0.00	-1.00
**GSDMB**	8.20	6.20	-2.00	-4.01	0.00	0.00	-1.00
**GPAT3**	10.43	8.40	-2.03	-4.09	0.00	0.00	-1.00
**PKP2**	7.81	5.76	-2.05	-4.14	0.00	0.01	-1.00
**RASSF6**	6.20	4.08	-2.12	-4.34	0.00	0.02	-1.00
**NWD2**	7.31	5.04	-2.27	-4.81	0.00	0.00	-1.00
**C6**	10.04	7.74	-2.30	-4.93	0.00	0.00	-1.00
**FAM95A**	6.42	4.09	-2.33	-5.02	0.00	0.00	-1.00
**COL6A6**	9.60	7.25	-2.35	-5.09	0.00	0.00	-1.00
**STOX1**	8.67	6.32	-2.35	-5.10	0.00	0.00	-1.00
**CA3**	10.47	8.02	-2.45	-5.46	0.00	0.00	-1.00
**RORB**	6.57	3.47	-3.11	-8.61	0.00	0.00	-1.00

Table S5 cont'd

**SPX**	9.35	6.12	-3.23	-9.37	0.00	0.00	-1.00
**SLC27A2**	6.94	3.56	-3.38	-10.42	0.00	0.00	-1.00
**RORB**	8.92	5.52	-3.40	-10.54	0.00	0.00	-1.00
**SLC27A2**	7.34	3.56	-3.78	-13.75	0.00	0.00	-1.00

Table S6 First 70 genes by Ethnicity

SYMBOL	lgFCH_Obese.AA.vs.EA	FCH_Obese.AA.vs.EA	pvals_Obese.AA.vs.EA	fdrs_Obese.AA.vs.EA	StatusFCH1.5FDR0.1_Obese.AA.vs.EA
SLC6A4	2.15	**4.43**	0.00	0.05	1.00
LINC01088	1.87	**3.65**	0.00	0.05	1.00
DUXAP10	1.82	**3.54**	0.00	0.05	1.00
DUXAP10	1.81	**3.50**	0.00	0.04	1.00
SIM1	1.70	**3.24**	0.00	0.05	1.00
DUXAP10	1.69	**3.22**	0.00	0.07	1.00
DUXAP10	1.69	**3.22**	0.00	0.07	1.00
CRYBB2	1.57	**2.98**	0.00	0.08	1.00
LINC01296	1.56	**2.94**	0.00	0.05	1.00
NOS1	1.51	**2.85**	0.00	0.04	1.00
POLR1A	1.48	**2.79**	0.00	0.03	1.00
ERAP2	1.43	**2.70**	0.00	0.06	1.00
KLRC2	1.38	**2.61**	0.00	0.07	1.00
COL8A1	1.36	**2.56**	0.00	0.05	1.00
B3GLCT	1.31	**2.47**	0.00	0.08	1.00
SLC6A4	1.26	**2.39**	0.00	0.05	1.00
LNPEP	1.25	**2.38**	0.00	0.05	1.00
CORIN	1.17	**2.26**	0.00	0.09	1.00
GALNT16	1.15	**2.23**	0.00	0.05	1.00
NDE1	1.14	**2.20**	0.00	0.06	1.00
C1QTNF1	1.11	**2.15**	0.00	0.03	1.00
EHD4	1.11	**2.15**	0.00	0.05	1.00
CACNB2	1.09	**2.14**	0.00	0.05	1.00
CENPBD1P1	1.09	**2.13**	0.00	0.05	1.00
SYDE1	1.08	**2.11**	0.00	0.08	1.00

Table S6 cont'd

**CCL8**	1.07	**2.10**	0.00	0.08	1.00
**ETV6**	1.07	**2.09**	0.00	0.04	1.00
**OASL**	1.04	**2.05**	0.00	0.07	1.00
**C8orf34-AS1**	1.01	**2.02**	0.00	0.08	1.00
**CSF1**	1.00	**2.00**	0.00	0.06	1.00
**EHD4**	0.98	**1.98**	0.00	0.08	1.00
**CACNB2**	0.97	**1.96**	0.00	0.04	1.00
**PTGER3**	0.95	**1.94**	0.00	0.10	1.00
**TCEA3**	0.96	**1.94**	0.00	0.08	1.00
**LINC00632**	0.94	**1.92**	0.00	0.05	1.00
**PYHIN1**	0.94	**1.92**	0.00	0.05	1.00
**HSPB6**	0.94	**1.92**	0.00	0.06	1.00
**C1QTNF2**	0.93	**1.91**	0.00	0.09	1.00
**FZD8**	0.93	**1.90**	0.00	0.08	1.00
**C10orf54**	0.93	**1.90**	0.00	0.03	1.00
**KIF6**	0.92	**1.89**	0.00	0.03	1.00
**NMNAT2**	0.89	**1.85**	0.00	0.06	1.00
**VSTM2L**	0.88	**1.85**	0.00	0.03	1.00
**GOLM1**	0.89	**1.85**	0.00	0.03	1.00
**MAP9**	0.88	**1.85**	0.00	0.05	1.00
**HSDL2**	0.88	**1.84**	0.00	0.07	1.00
**TCEANC2**	0.88	**1.84**	0.00	0.04	1.00
**MGST1**	0.88	**1.84**	0.00	0.10	1.00
**GARNL3**	0.88	**1.84**	0.00	0.07	1.00
**LOC101927648**	0.86	**1.82**	0.00	0.06	1.00
**ZNF555**	0.87	**1.82**	0.00	0.08	1.00
**LTBP2**	0.86	**1.82**	0.00	0.06	1.00
**PAPPA**	0.86	**1.81**	0.00	0.08	1.00
**ACVRL1**	0.85	**1.81**	0.00	0.05	1.00
**USP42**	0.86	**1.81**	0.00	0.10	1.00
**CFP**	0.86	**1.81**	0.00	0.08	1.00
**ZNF683**	0.86	**1.81**	0.00	0.08	1.00
**RPP25**	0.85	**1.80**	0.00	0.05	1.00
**KCNJ12**	0.83	**1.78**	0.00	0.08	1.00
**MMAB**	0.83	**1.77**	0.00	0.03	1.00
**CYTH4**	0.82	**1.77**	0.00	0.07	1.00
**RDH16**	0.82	**1.77**	0.00	0.07	1.00
**ATP6V0E2**	0.82	**1.77**	0.00	0.09	1.00
**ZHX2**	0.81	**1.76**	0.00	0.05	1.00

Table S7

Last 70 genes by Ethnicity

SYMBOL	lgFCH_Obese.AA.vs.EA	FCH_Obese.AA.vs.EA	pvals_Obese.AA.vs.EA	fdrs_Obese.AA.vs.EA	StatusFCH1.5FDR0.1_Obese.AA.vs.EA
**PTGR2**	-1.27	**-2.41**	0.00	0.10	-1.00
**DNAJB14**	-1.28	**-2.42**	0.00	0.08	-1.00
**SLC1A6**	-1.29	**-2.44**	0.00	0.05	-1.00
**ZCCHC11**	-1.28	**-2.44**	0.00	0.05	-1.00
**SRSF1**	-1.29	**-2.45**	0.00	0.04	-1.00
**OGT**	-1.31	**-2.47**	0.00	0.07	-1.00
**C5orf24**	-1.31	**-2.49**	0.00	0.07	-1.00
**MLLT10**	-1.31	**-2.49**	0.00	0.07	-1.00
**LOC101927391**	-1.31	**-2.49**	0.00	0.08	-1.00
**RPRD1A**	-1.32	**-2.50**	0.00	0.07	-1.00
**MBNL1**	-1.33	**-2.51**	0.00	0.07	-1.00
**RASSF5**	-1.34	**-2.53**	0.00	0.10	-1.00
**THUMPD3-AS1**	1.34	**-2.53**	0.00	0.06	-1.00
**BLOC1S5**	-1.34	**-2.54**	0.00	0.09	-1.00
**WDR72**	-1.35	**-2.54**	0.00	0.04	-1.00
**SGPP1**	-1.36	**-2.57**	0.00	0.07	-1.00
**THUMPD3-AS1**	-1.37	**-2.59**	0.00	0.05	-1.00
**B3GNT2**	-1.39	**-2.63**	0.00	0.09	-1.00
**DENR**	-1.40	**-2.63**	0.00	0.05	-1.00
**FRG1JP**	-1.41	**-2.65**	0.00	0.05	-1.00
**LOC284578**	-1.42	**-2.67**	0.00	0.06	-1.00
**GATM**	-1.42	**-2.68**	0.00	0.09	-1.00
**TSC22D1**	-1.43	**-2.70**	0.00	0.06	-1.00

Table S7 cont'd

**LOC283788**	-1.44	**-2.72**	0.00	0.07	-1.00
**MFSD8**	-1.45	**-2.73**	0.00	0.06	-1.00
**NUP50**	-1.45	**-2.74**	0.00	0.04	-1.00
**TMED2**	-1.46	**-2.75**	0.00	0.10	-1.00
**INO80D**	-1.46	**-2.75**	0.00	0.06	-1.00
**PPP4R4**	-1.46	**-2.76**	0.00	0.07	-1.00
**AP4E1**	-1.47	**-2.77**	0.00	0.08	-1.00
**RRN3P1**	-1.47	**-2.78**	0.00	0.03	-1.00
**LYZ**	-1.49	**-2.81**	0.00	0.10	-1.00
**CRYBB2P1**	-1.53	**-2.89**	0.00	0.09	-1.00
**ANLN**	-1.54	**-2.91**	0.00	0.05	-1.00
**FCGR3B**	-1.54	**-2.92**	0.00	0.03	-1.00
**CCNG2**	-1.57	**-2.97**	0.00	0.09	-1.00
**RSRP1**	-1.57	**-2.97**	0.00	0.06	-1.00
**CEACAM5**	-1.58	**-2.99**	0.00	0.09	-1.00
**SMARCA2**	-1.61	**-3.04**	0.00	0.09	-1.00
**RICTOR**	-1.61	**-3.05**	0.00	0.08	-1.00
**YWHAZ**	-1.63	**-3.09**	0.00	0.07	-1.00
**UBE2Z**	-1.64	**-3.12**	0.00	0.05	-1.00
**SULT1E1**	-1.66	**-3.15**	0.00	0.05	-1.00
**PRPF39**	-1.66	**-3.16**	0.00	0.05	-1.00
**TMED2**	-1.69	**-3.23**	0.00	0.07	-1.00
**SULT1E1**	-1.72	**-3.30**	0.00	0.03	-1.00
**NFIA**	-1.79	**-3.46**	0.00	0.08	-1.00
**SOX9**	-1.96	**-3.89**	0.00	0.10	-1.00
**CFTR**	-1.99	**-3.98**	0.00	0.03	-1.00
**UBA6**	-2.06	**-4.16**	0.00	0.07	-1.00
**GABRP**	-2.07	**-4.19**	0.00	0.03	-1.00
**FMO2**	-2.13	**-4.37**	0.00	0.07	-1.00
**CDH11**	-2.32	**-4.98**	0.00	0.05	-1.00
**SCGB2A2**	-2.60	**-6.04**	0.00	0.10	-1.00

Supplemental Figure S1

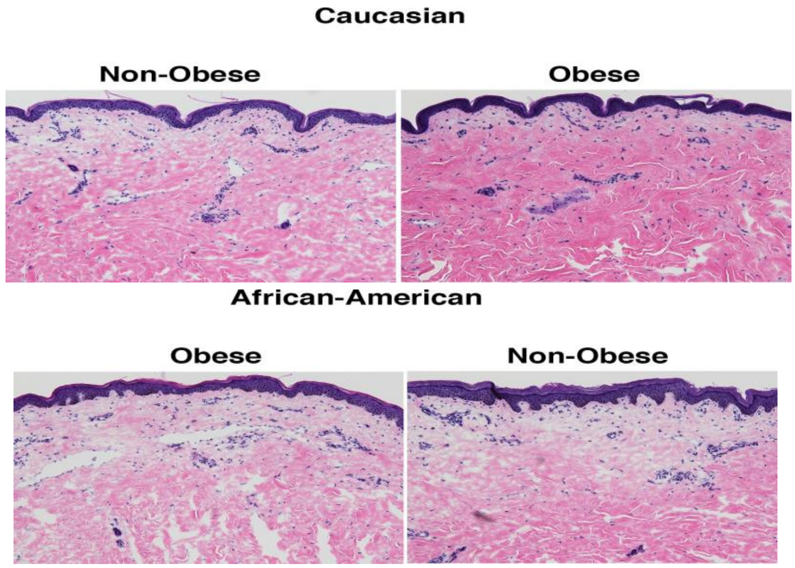

Epidermal Thickness in Obese and Non-Obese Subjects

	n	Mean	+/-SD	*p*=
		microns		
Obese	10	45.1	11.5	0.512
Non-Obese	10	41.8	9.8	
Obese and non-obese				
African Americans	8	48.4	7.9	0 .086
Obese and non-obese				
European Americans	12	40.1	11.1	

Epidermal thickness measured as microns, mean +/-SD, and difference (p values) in the number (n) of obese and non-obese subjects; obese and non-obese and African American subjects and obese and non-obese European American subjects is shown.

Supplemental Figure S2

SKIN
Obese vs. Non-Obese

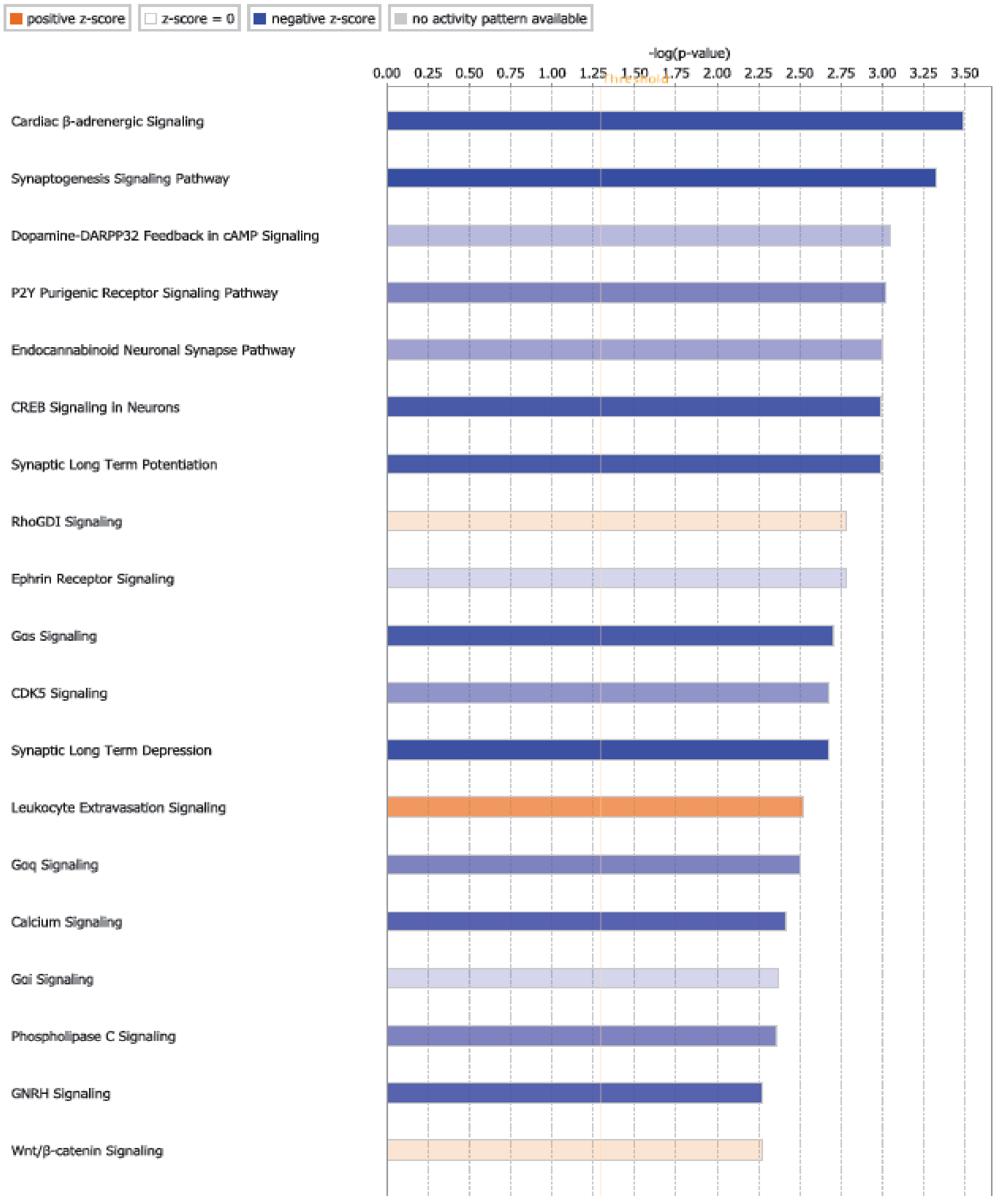

Supplemental Figure S3

FAT: Obese vs. Non-Obese

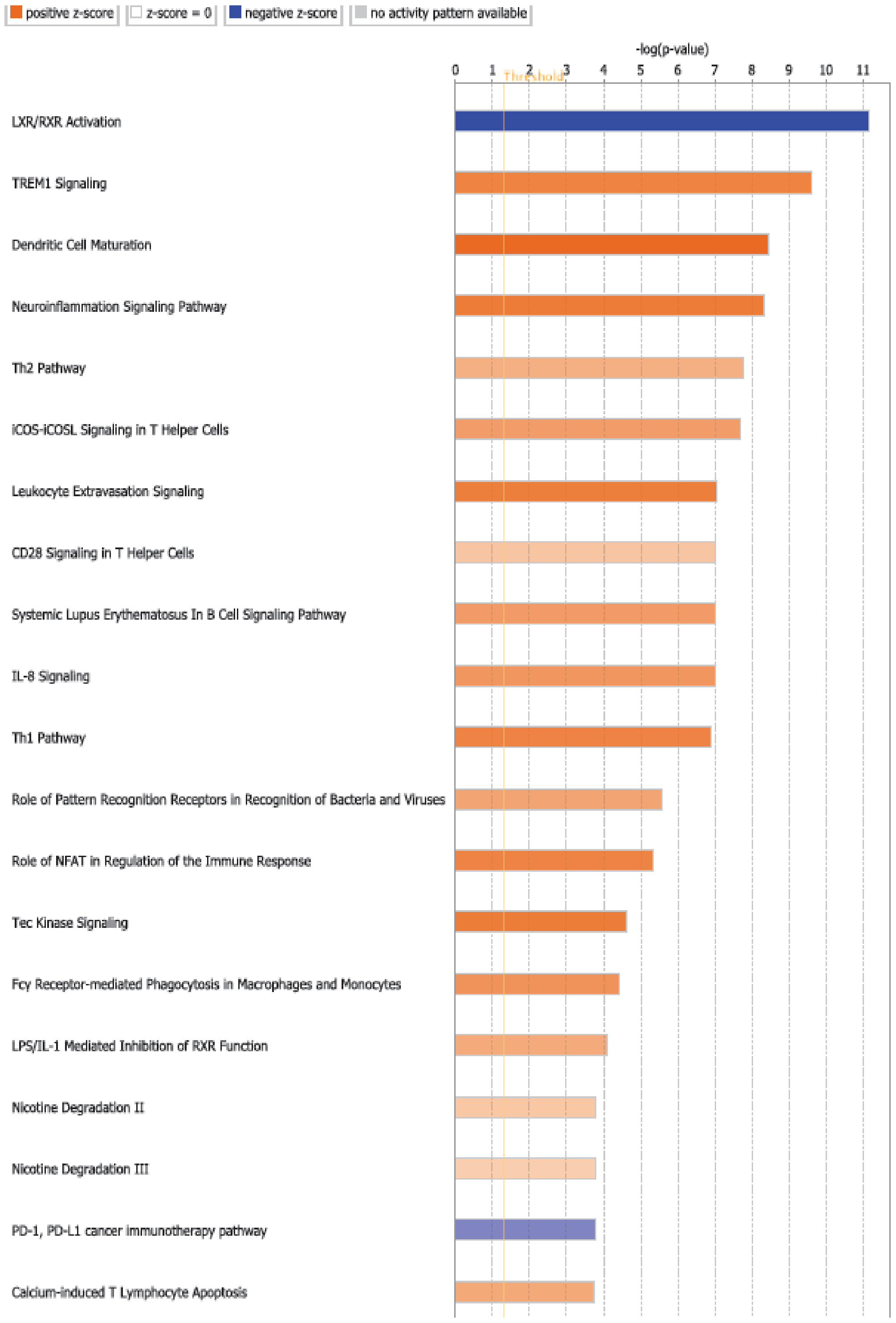

Supplemental Figure S4

SKIN: Obese African American vs European American

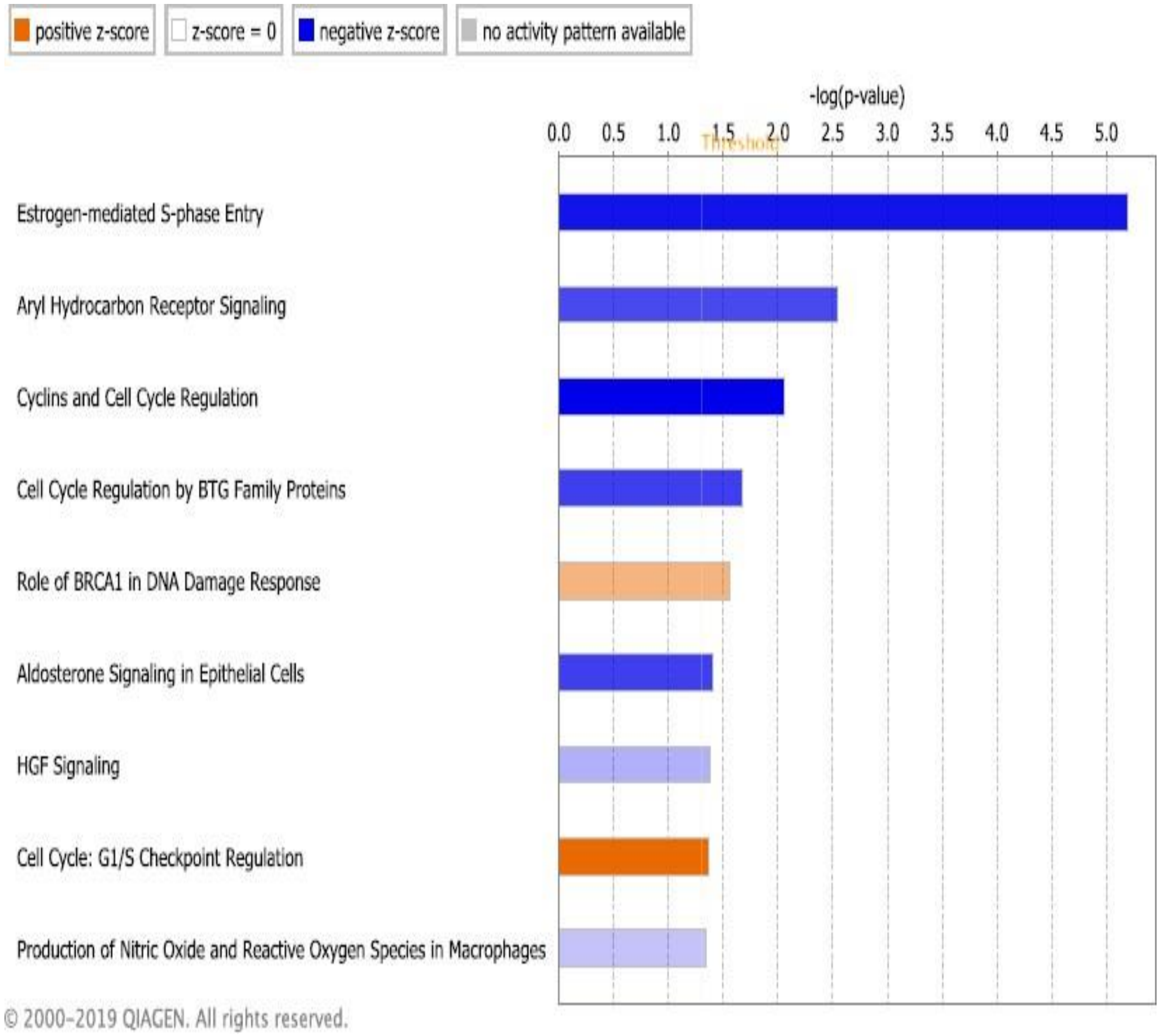

Supplemental Figure S5

FAT: Obese African American vs. European American

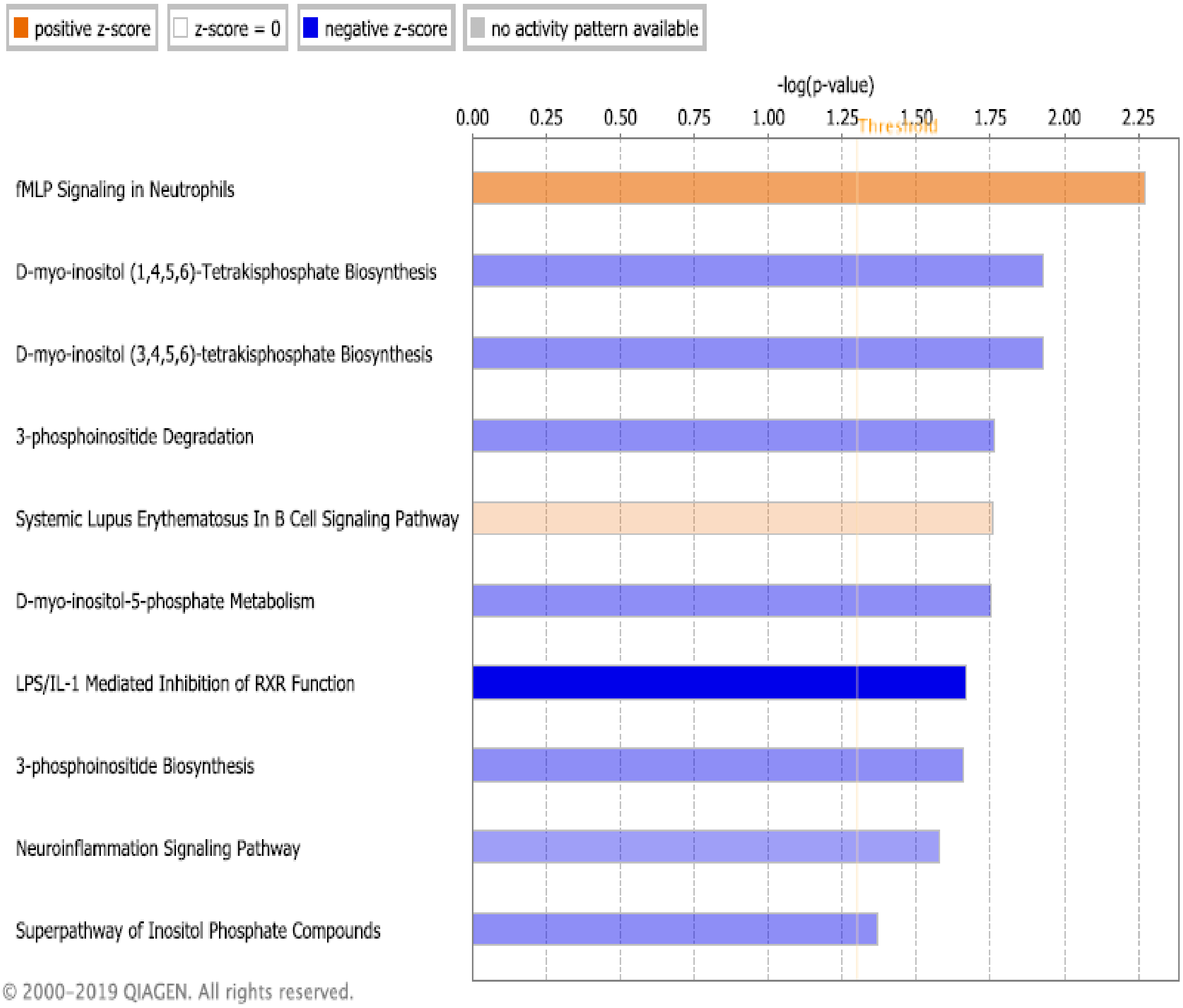

© 2000-2019 QIAGEN. All rights reserved.

Supplemental Figure S6

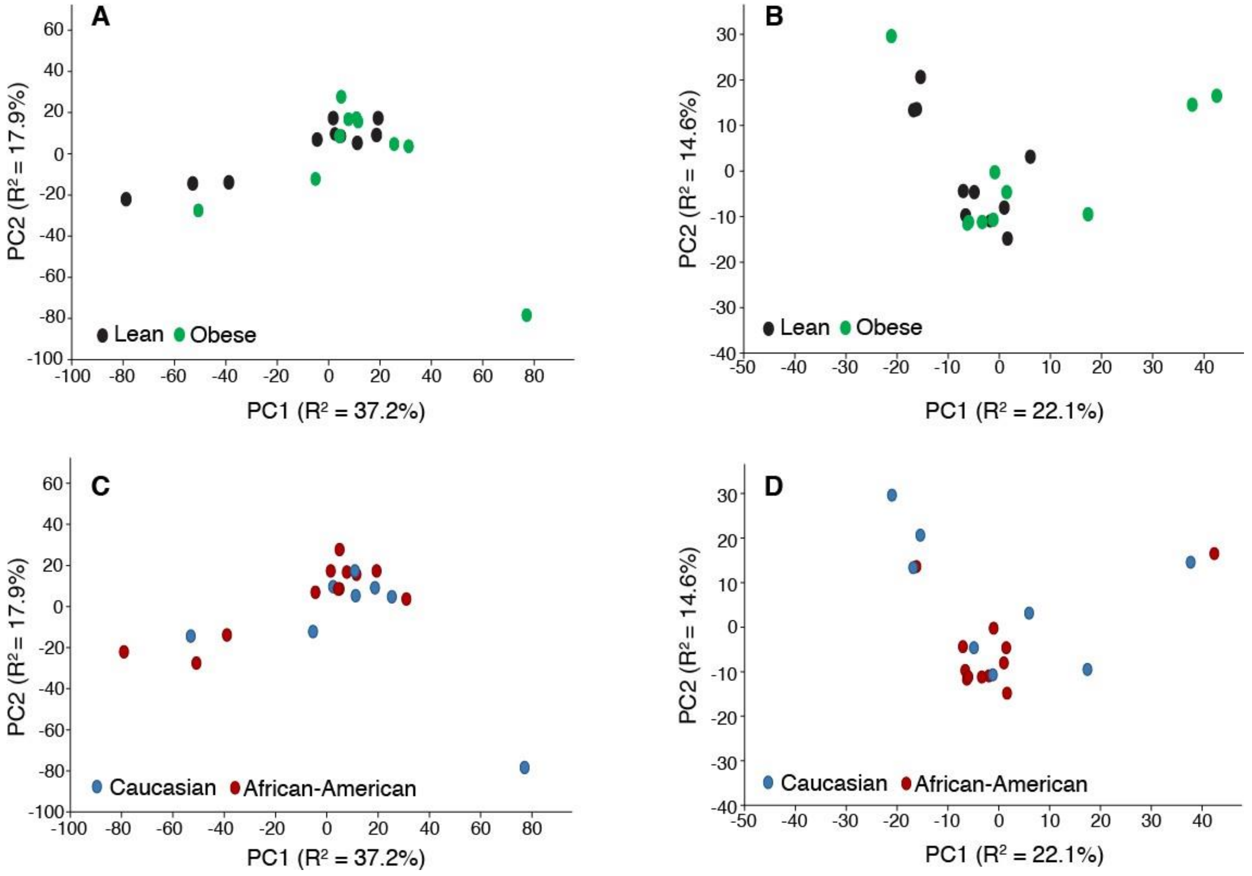

Supplemental Figure Legends

Supplemental Figure S1

A. Typical photomicrograph of skin biopsies from obese and non-obese subjects and from African American and European American subjects

   Paired by age and skin colour

B. Dimensions of the epidermis of obese and non-obese African American and European American subjects.

   Note no difference in epidermal thickness between obese and non-obese subjects but a trend to a wider epidermis in African Americans than in European American subjects.

Supplemental Figure S2

Gene expression pathway analysis between the skin of obese and non-obese subjects.

Data shown from analysis of all differentially expressed genes.

Log value shown in blue = negative *z* score

Log value shown in orange = positive *z* score.

Supplemental Figure S3

Gene expression pathway analysis between the subdermal fat of obese and non-obese subjects.

Data shown from analysis of all differentially expressed genes.

Log value shown in blue = negative *z* score

Log value shown in orange = positive *z* score.

Supplemental Figure S4

Gene expression pathway analysis between the skin of obese African American and obese European American subjects.

Data shown from analysis of all differentially expressed genes.

Log value shown in blue = negative *z* score

Log value shown in orange = positive *z* score.

Supplemental Figure S5

Gene pathway analysis between the subdermal fat of obese African American and obese European American subjects.

Data shown from analysis of all differentially expressed genes.

Log value shown in blue = negative *z* score

Log value shown in orange = positive *z* score.

Supplemental Figure S6

PCA models comparing the metabolic content of swab samples collected from obese and non-obese (A, positive ionization mode; B, negative ionization mode), and European American (labeled Caucasian) and African American individuals (C, positive ionization mode; D, negative ionization mode). Metabolic profiles were measured by UPLC-MS.
